# Isolated IgG4-related Infundibulo-hypophysitis

**DOI:** 10.1210/jcemcr/luae182

**Published:** 2024-10-16

**Authors:** Margaret E Allen, Ryan T Beck, Nathan T Zwagerman, Dylan Coss, Amy Fisco, Adriana G Ioachimescu

**Affiliations:** Medical College of Wisconsin, Milwaukee, WI 53226 USA; Department of Radiology, Medical College of Wisconsin, Milwaukee, WI 53226 USA; Department of Neurosurgery, Medical College of Wisconsin, Milwaukee, WI 53226 USA; Department of Pathology and Laboratory Medicine, Medical College of Wisconsin, Milwaukee, WI 53226 USA; Department of Medicine, Division of Endocrinology, Ascension All Saints Hospital, Racine, WI, USA; Department of Neurosurgery, Medical College of Wisconsin, Milwaukee, WI 53226 USA; Department of Medicine, Division of Endocrinology, Medical College of Wisconsin, Milwaukee, WI 53226 USA

**Keywords:** hypophysitis, hypopituitarism, IgG4 disease, pituitary mass

## Abstract

A 72-year-old man presented with several months of weakness, poor appetite, and depressed moods. Laboratory tests indicated central hypocortisolism, hypothyroidism and hypogonadism, and mild hyperprolactinemia. Imaging indicated a homogenously enhancing solid suprasellar mass inseparable from the hypothalamus and contiguous with a thickened proximal infundibulum. Neuro-ophthalmological evaluation was normal. Symptoms improved with hydrocortisone, levothyroxine, and testosterone replacement. After 6 months, transsphenoidal biopsy was performed due to mass enlargement and revealed fibrosis, lymphoplasmacytic infiltration, and CD138 and IgG4 staining. The levels of serum IgG4, complement, inflammatory markers, protein electrophoresis, amylase, and lipase and imaging of the chest, abdomen, and thyroid were unremarkable. After 1 month of prednisone therapy (starting dose 40 mg/day), the mass significantly involuted and remained stable afterward. Prednisone was gradually tapered to 5 mg daily over 10 weeks. During 22 months of follow-up, no systemic IgG4 disease was detected. Glucocorticoid, thyroid, and testosterone replacement was continued. This case of isolated IgG4-related hypophysitis illustrates the variable presentation that may not entail vasopressin deficiency or clinical mass effect. This entity should be considered in the differential diagnosis of suprasellar masses even in the absence of IgG4 systemic disease or characteristic serology. Management entails multidisciplinary collaboration and long-term follow-up.

## Introduction

IgG4-related disease (IgG4-RD) is an immune-mediated condition characterized by lymphoplasmacytic infiltration, storiform fibrosis, and obliterative phlebitis (1). IgG4-RD can affect multiple organs, with most frequent presentations entailing pancreatitis, sclerosing cholangitis, bilateral sialadenitis, retroperitoneal fibrosis, and/or orbital inflammatory disease (2). Also, eye involvement from IgG4-RD can mimic Graves' ophthalmopathy (3). IgG4-RD was first recognized as a unified disease in the early 2000s based on characteristic pathology and serology. In a claims-based database study from the United States, IgG4-RD incidence increased from .78 to 1.39 per 100 000 person-years in 2015 compared to 2019 (4). Clinical presentation is usually subacute or chronic with a wide spectrum from mild localized symptoms to major tissue damage and even organ failure. Its pathogenesis is incompletely understood and is currently attributed to abnormal immune responses mediated by T-helper type 2 cells, regulatory T cells, and CD4+ cytotoxic T lymphocytes with increased production of interleukins and activation of B cells to plasma cells that produce IgG4 and IgE antibodies. Serum IgG4 levels are often increased >135 mg/dL (9.01 µmol/L) (normal reference ranges are laboratory-specific, with an upper normal range below this value), along with hypocomplementemia, which are monitored during treatment. Early recognition of IgG4-RD is important to avoid permanent organ damage. Treatment with glucocorticoids (GC) is usually effective, especially in the inflammatory and proliferative stages; however, IgG4-RD is a remitting-relapsing disease that can flare after GC taper (5).

Pituitary gland involvement is rare in IgG4-RD, reported in up to 8% of cases (6). The first case report of IgG4-related hypophysitis (IgG4-RH) was in 2004 and the first histopathology confirmation in 2007 (7, 8). Leporati criteria are used for diagnosis as follows: (1) pituitary histopathology (criterion #1, gold standard), (2) a combination of suggestive pituitary magnetic resonance imaging (MRI) findings and nonpituitary histopathology (criteria 2 + 3), or (3) suggestive pituitary MRI findings and positive IgG4 serology and GC response (criteria 2 + 4 + 5) (Table 1) (9).

**Table 1.** Leporati diagnostic criteria for IgG4-RH (9)

(1) Pituitary histopathology with >10 IgG4-positive cells/high-power field
(2) Pituitary MRI with sellar mass or thickened pituitary stalk
(3) Biopsy-proven IgG4-positivity in other organs
(4) Serology with increased serum IgG4
(5) Response to glucocorticoids

Abbreviations: IgG4-RH, IgG4-related hypophysitis; MRI, magnetic resonance imaging.

IgG4-RH is a relatively recent and rare occurrence in endocrine clinical practice. Differentiation from other inflammatory pituitary disorders can be challenging. The long-term natural course, including the response to GC treatment, is incompletely understood. In this article, we present a case of isolated IgG4-RH with histopathological confirmation and negative serology with a follow-up of 22 months.

## Case Presentation

A 72-year-old Caucasian, non-Hispanic man presented in August 2022 with several months of muscle weakness, fatigue, poor appetite, hot flashes, and depressed mood. Involuntary weight loss started approximately 2 years previously. A review of systems was remarkable for low libido and bilateral tinnitus. Notably, headaches, vision changes, polyuria, or polydipsia were absent. Family history was remarkable for type 2 diabetes mellitus and negative for autoimmune disease. Past medical history included B12 deficiency, overactive urinary bladder, and benign prostatic hyperplasia, which was previously treated with laser therapy. Medications were cyanocobalamin and mirabegron ER. On examination, blood pressure was 110/74, pulse 83, weight 197 lbs, and height 6'1” (body mass index 26.0 kg/m2). Signs of hormone overproduction and neurological abnormalities were absent. Gynecomastia was absent. Body hair was decreased. A recent urological examination done prior to presentation to endocrinology indicated normal external genitalia.

## Diagnostic Assessment

**Figure 1.**
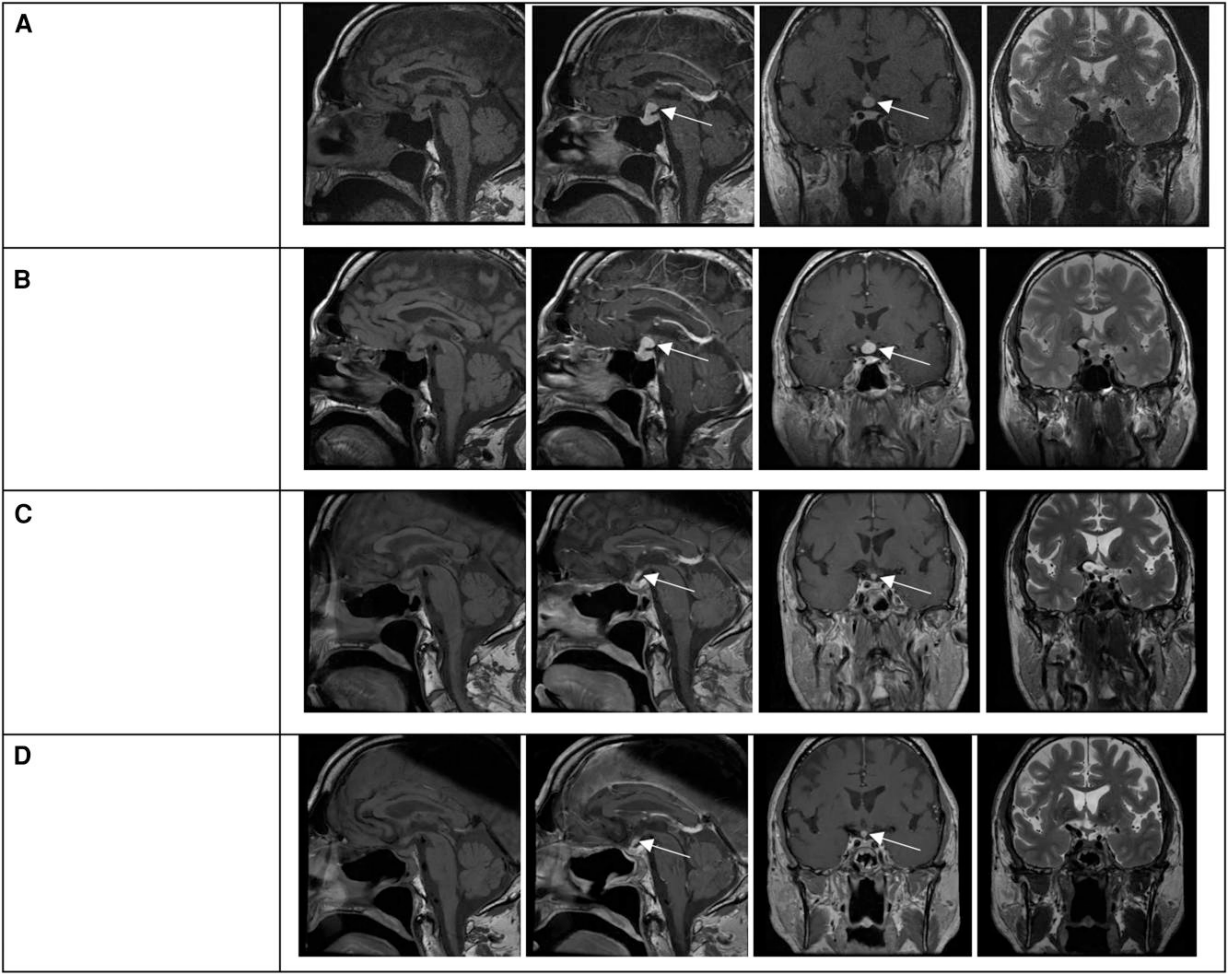
Left to right: noncontrast sagittal T1-weighted, postcontrast sagittal T1-weighted, postcontrast coronal T1-weighted, and noncontrast coronal T2-weighted. White arrows denote the suprasellar lesion in the images where it is best visualized. (A) At presentation August 2022; (B) before biopsy March 2023; (C) 1 month after starting 40 mg/day prednisone August 2023; (D) after 10 months of prednisone 5 mg/day June 2024.

Hemogram, differential white blood cells, and chemistry panel were unremarkable. Hormone evaluation revealed ACTH, TSH, FSH, and LH deficiencies and mild hyperprolactinemia (Table 2). Sellar MRI demonstrated a .8 × .7 × .8 cm suprasellar mass inseparable from the hypothalamus contiguous with a thickened pituitary stalk and with mass effect upon the optic chiasm (Fig. 1A). Neuro-ophthalmology evaluation was unremarkable.

**Table 2.** Endocrine laboratory results

Date	August 2022	October 2023	February 2024	June 2024
**Laboratory parameter, standard range**	**Laboratory data,** **conventional units** **(SI)**			
IGF-1, 25-242 ng/mL (μg/L)	29 ng/mL (3.79 μg/L)	84 ng/mL (10.98 μg/L)	95 ng/mL (12.42 μg/L)	110 ng/mL (14.38 μg/L)
Prolactin, 3.46-19.40 ng/mL (μg/L)	50.89 ng/mL (50.89 μg/L)	62.50 ng/mL (62.50 μg/L)	69.70 ng/mL (69.70 μg/L)	74.70 ng/mL (74.70 μg/L)
Am Cortisol, 4.80-19.50 μg/dL (132.48-538.20 nmol/L)	<1.0 μg/dL (<27.6 nmol/L)			4.50 μg/dL*^a^* (124.2 nmol/L*^a^*)
ACTH, 7.20-63.30 pg/mL (ng/L)	6.80 pg/mL (6.80 ng/L)			20.20 pg/mL*^a^* (20.20 ng/L*^a^*)
Testosterone, 240-950 ng/dL (8.33-32.97 nmol/L)	7.0 ng/dL (.24 nmol/L)	783.5 ng/dL*^b^* (27.18 nmol/L*^b^*)	1222 ng/dL*^b^* (42.40 nmol/L*^b^*)	998 ng/dL*^b^* (34.60 nmol/L*^b^*)
LH, .57-12 mIU/mL (IU/L)	.16 mIU/mL (.16 IU/L)	<.12 mIU/mL (<.12 IU/L)		
FSH, 1-12 mIU/mL (IU/L)	1.5 mIU/mL (1.5 IU/L)	.70 mIU/mL (.70 IU/L)		
Free T4, .70-1.48 ng/dL (9.03-19.10 pmol/L)	.63 ng/dL (8.13 pmol/L)	1.16 ng/dL*^c^* (14.96 pmol/L*^c^*)	1.10 ng/dL*^c^* (14.19 pmol/L*^c^*)	1.35 ng/dL*^c^* (17.42 pmol/L*^c^*)
TSH, .35-4.94 μIU/mL (mIU/L)	.20 μIU/mL (.20 mIU/L)	<.001 μIU/mL*^c^* (<.001 mIU/L*^c^*)		.01 μIU/mL (.01 mIU/L)

Values in parentheses are System of Units (SI).

^*a*^Before taking 5 mg prednisone.

^*b*^On testosterone replacement.

^*c*^On levothyroxine replacement.

## Treatment

After neurosurgery consultation, imaging surveillance was recommended given absent clinical evidence of mass effect and possible surgical risks. The patient was initiated on hormone replacement with hydrocortisone 10 mg (5 mg in the morning and 5 mg in the afternoon) and levothyroxine 50 mcg daily. During follow-up, hydrocortisone morning dose was increased to 12.5 mg and levothyroxine dose to 75 mcg daily; also, testosterone cypionate injections intramuscularly 100 mg every 14 days were initiated. The patient reported an overall improvement in his health and no changes in thirst or urination.

**Figure 2.**
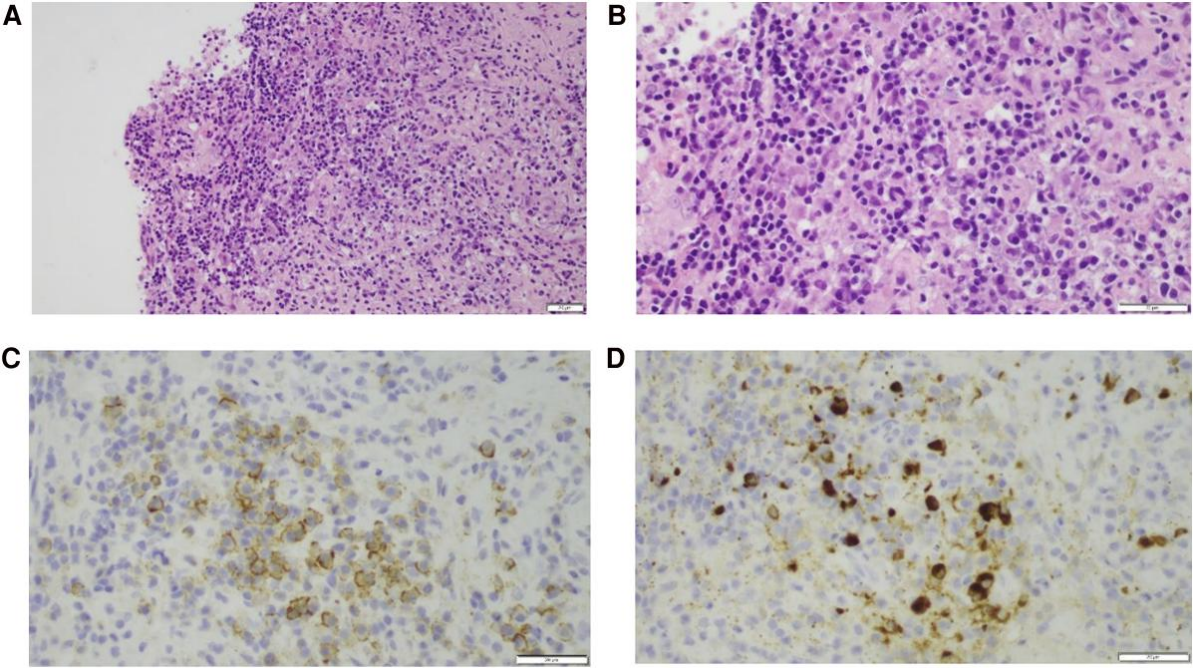
(A) H&E 20 × with marked lymphocytic infiltrate with prominent plasma cells in a background of fibrosis; (B) H&E 40 × with marked lymphocytic infiltrate with prominent plasma cells in a background of fibrosis; (C) immunohistochemistry for CD138, plasma cell marker (positive membranous staining); (D) immunohistochemistry for IgG4, positive in the majority of plasma cells. Abbreviation: H&E, hematoxylin and eosin staining.

After 6 months (March 2023), imaging showed an increase of the suprasellar mass to 1.2 × .8 × 1.1 cm. A transsphenoidal mass biopsy was performed in May 2023. Histopathological exam demonstrated fibrosis and a chronic inflammatory infiltrate composed of T-cells, histiocytes, and IgG4-positive plasma cells (Fig. 2). Rheumatology evaluation was performed, with negative blood, urine, and imaging tests (computed tomography chest and abdomen, thyroid ultrasound) for other organ involvement. Serum IgG4 was normal, with a level of .94 g/L (6.27 µmol/L) (normal reference range: .01-1.23 g/L, .07-8.21 µmol/L). Complement levels were normal: C3 was 1.44 g/L (9.61 µmol/L) (normal reference range: .9-1.8 g/L, 6.00-12.01 µmol/L) and C4 was .26 g/L (1.74 µmol/L) (normal reference range: .1-.4 g/L, .67-2.67 µmol/L). The erythrocyte sedimentation rate was 23 (normal range: 0-32 mm/hr) and C reactive protein was undetectable at <5 mg/L (<47.62 nmol/L).

In July 2023, IgG4-RH treatment was initiated with prednisone 40 mg daily and hydrocortisone was stopped. Prednisone was decreased in 10 mg decrements every 2 weeks for 6 weeks, then in 5 mg increments until a 5 mg daily dose was attained (total duration of taper was 10 weeks). Thyroid and testosterone replacement were continued. MRI obtained 1 month after starting prednisone (August 2023) showed decreased mass size and decreased thickening of the infundibulum (Fig. 1C). MRIs done in December 2023 (not shown) and June 2024 (Fig. 1D) were stable.

## Outcome and Follow-up

At the last visit in June 2024, the patient reported doing well except for his inability to lose weight (he previously had gained 16 lbs while on a higher dose of prednisone) and new heartburn for which he started omeprazole 20 mg daily with good results. He was taking a physiologic prednisone dose of 5 mg daily, levothyroxine 88 mcg daily, and testosterone cypionate injections intramuscularly 100 mg every 2 weeks. On examination, his blood pressure was 118/78, pulse 69, weight 212 lbs, body mass index 29 kg/m^2^, and body surface area 2.21 m^2^. There were no signs of cortisol excess or optic neuropathy. Serum levels of IgG4, complement, inflammatory markers were normal. After reviewing hormone levels, the testosterone dose was decreased to 75 mg every 2 weeks. There was a rise in insulin-like growth factor levels during GC treatment (Table 2). Given his improved Am serum cortisol and ACTH levels right before taking his prednisone dose, replacing prednisone with an equivalent dose of hydrocortisone (15 mg in Am and 5 mg in Pm) was recommended with plans to recheck his Am cortisol level after 1 month, repeat neuro-ophtalmological and IgG4-RD serological evaluation every 3 months, and repeat imaging after 6 months.

## Discussion

We present a case of apparent isolated IgG4-RH presenting with multiple anterior pituitary deficiencies and a suprasellar mass close to the hypothalamus. The differential diagnosis of suprasellar masses includes tumors (craniopharyngioma, pituicytoma, germinoma, metastases, pituitary adenomas), infiltrative disorders (sarcoidosis, histiocytic disorders, Wegener granulomatosis), infections (human immunodeficiency virus, fungal, tuberculosis), and autoimmune hypophysitis. Primary autoimmune hypophysitis is the most frequent type of hypophysitis. In recent years, the prevalence of secondary hypophysitis increased in the context of immunotherapy for malignancies (10). IgG4-RH can occur with or without systemic IgG4-RD manifestations. A histopathological reevaluation of 29 samples previously identified as primary autoimmune hypophysitis from the German Pituitary Tumor Registry reclassified 12 specimens (41.4%) as IgG4-RH (11). Indeed, differentiation is not possible based on radiological appearance or pituitary hormone tests; also, lymphocytic hypophysitis and IgG4-RH have similar histopathological features, except for IgG4 plasma cell infiltrate. The German study findings and increasing reports of IgG4-RH indicate its prevalence is probably underestimated.

Our PubMed literature review identified 143 IgG4-RH cases, of which 116 were summarized in a review from 2021 (6). We identified an additional 27 cases (12-17) and reviewed available information regarding clinical presentation, pituitary hormone abnormalities, diagnostic modalities, clinical course, and treatment. Men were more frequently affected (67.4%) than women (32.6%). In the Amirbaigloo et al review, 50% of patients were older than 56 (age range 14-87) and men were older than women (median age 62 vs 38 in women) (6). Clinical presentation entailed combined anterior hypopituitarism (75.0%), vasopressin deficiency (61.8%), and/or clinical evidence of mass effect (headaches 31.9%, vision changes 43.9%). Hyperprolactinemia was detected in 40.9% of cases. A minority of patients had normal pituitary function. In our case, presentation entailed anterior hypopituitarism without clinical signs of vasopressin deficiency or hypernatremia. Also, our patient did not develop polyuria after initiation of hydrocortisone replacement or high-dose prednisone. This makes partial vasopressin deficiency unmasked by GC administration unlikely (18). In a single-center study of 170 patients with hypopituitarism and/or vasopressin deficiency, after exclusion of other known causes, 32 were screened for IgG4-RH. The diagnosis was confirmed in 7 patients, accounting for 30% of patients presenting with hypophysitis, 22% of patients with hypopituitarism/vasopressin deficiency of unknown cause, and 4% of all patients with hypopituitarism/vasopressin deficiency. Of the 7 patients, 4 had multiple coexisting IgG4-RD involvement (19). In a single-center study of 10 patients with IgG4-RH, an average of 3 extra-pituitary organs were involved (12). These studies highlight the importance of screening for IgG4-RH in the appropriate clinical scenarios, ie, hypophysitis and other organ involvement (synchronous or metachronous).

In our literature review, elevated serum IgG4 levels occurred in 69.7% of IgG4-RH cases in which testing was performed. Normal IgG4 levels can occur in patients with IgG4-RD; these antibodies are not considered pathogenic but rather an epiphenomenon. In our literature review, isolated IgG4-RH was reported in 30.6% of cases, and pituitary biopsy was performed in 50.7%. The diagnosis is especially challenging in patients with isolated IgG4-RH and negative serology (ie, normal immunoglobulin and complement levels), like in our case. Conversely, mild elevations in plasma IgG4 levels may be found in other disorders (20).

Management of IgG4-RH can entail 1 of the following: high-dose GC therapy or close monitoring along with replacement of hormone deficits—the latter in the absence of mass effect or enlargement on serial imaging. Like other forms of IgG4-RD, IgG4-RH is characterized by a rapid response to GC, with 1 or more of the following outcomes: improved clinical mass effect, decreased lesion size, decreased IgG4 levels, and increased complement levels. For IgG4-RD, the initial recommended dose of prednisone or prednisolone is .6/kg/day (30-40 mg) with a subsequent taper over 3 months (1), which was the regimen chosen for our patient. From our literature review, the mean starting dose of prednisone for IgG4-RH was 47 mg/day. A minority of cases responded to physiologic GC replacement doses (21). In our case, however, radiological progression occurred initially on physiologic doses of hydrocortisone, which prompted the transsphenoidal biopsy. IgG4-RH relapse was documented in some reported cases during taper, usually at doses of 10 to 15 mg prednisone/day. GC-sparing medications were used with variable success in this situation, including rituximab, azathioprine, mycophenolate mofetil, and others (6). Long-term data regarding the optimal duration of high-dose GC therapy, endurance of GC effect, and reversibility of the pituitary hormone deficits are lacking. In the reported cases where pituitary function recovery was documented, it was usually incomplete (6, 12). In our case, IgG4-RH had a durable response to prednisone despite tapering to physiologic doses after 2.5 months. At the last clinic visit, laboratory tests indicated partial recovery of the ACTH axis. The patient was interested in stopping the prednisone to help with his weight loss efforts and heartburn. Therefore, prednisone was replaced with equivalent doses of hydrocortisone, with plans to follow up closely for IgG4-RH clinical, serological, and radiological recurrence and to revisit recovery of other pituitary axes at future visits.

In summary, IgG4-RH prevalence, prognosis, and long-term response to therapy of IgG4-RH require further understanding. Due to its possible association with other organ involvement, a thorough clinical evaluation and serological tests are important in patients with hypophysitis and idiopathic hypopituitarism. A multicentric prospective study in patients with hypophysitis is necessary to unveil the spectrum of IgG4-RH and provide guidelines for standardized management.

## Learning Points

- IgG4-RH has variable clinical presentation, and diagnosis requires a high index of clinical suspicion.
- Serum IgG4 should be measured in patients with hypophysitis and idiopathic hypopituitarism.
- IgG4-RH has a characteristic prompt response to 30 to 40 mg of prednisone.
- The prevalence and long-term course of IgG4-RH require further study.

## Data Availability

Original data generated and analyzed during this study are included in this published article.
